# Determinants of Fast Food Consumption among Iranian High School Students Based on Planned Behavior Theory

**DOI:** 10.1155/2013/147589

**Published:** 2013-07-11

**Authors:** Gholamreza Sharifirad, Parastoo Yarmohammadi, Leila Azadbakht, Mohammad Ali Morowatisharifabad, Akbar Hassanzadeh

**Affiliations:** ^1^Department of Health Education and Health Promotion, Isfahan University of Medical Sciences, Isfahan, Iran; ^2^Kashani Hospital, Shahrekord University of Medical Science, Shahrekord, Iran; ^3^Food Security Research Center, Department of Community Nutrition, School of Health & Nutrition, Isfahan University of Medical Sciences, Isfahan, Iran; ^4^Department of Health Education and Health Promotion, Yazd Shahid Sadoughi University of Medical Sciences, Yazd, Iran; ^5^Department of Statistics and Epidemiology, School of Health, Isfahan University of Medical Sciences, Isfahan, Iran

## Abstract

*Objective*. This study was conducted to identify some factors (beliefs and norms) which are related to fast food consumption among high school students in Isfahan, Iran. We used the framework of the theory planned behavior (TPB) to predict this behavior. *Subjects & Methods*. Cross-sectional data were available from high school students (*n* = 521) who were recruited by cluster randomized sampling. All of the students completed a questionnaire assessing variables of standard TPB model including attitude, subjective norms, perceived behavior control (PBC), and the additional variables past behavior, actual behavior control (ABC). *Results*. The TPB variables explained 25.7% of the variance in intentions with positive attitude as the strongest (*β* = 0.31, *P* < 0.001) and subjective norms as the weakest (*β* = 0.29, *P* < 0.001) determinant. Concurrently, intentions accounted for 6% of the variance for fast food consumption. Past behavior and ABC accounted for an additional amount of 20.4% of the variance in fast food consumption. *Conclusion*. Overall, the present study suggests that the TPB model is useful in predicting related beliefs and norms to the fast food consumption among adolescents. Subjective norms in TPB model and past behavior in TPB model with additional variables (past behavior and actual behavior control) were the most powerful predictors of fast food consumption. Therefore, TPB model may be a useful framework for planning intervention programs to reduce fast food consumption by students.

## 1. Introduction

Fast food intake is still increasing specially among younger generation [1]. Fast food intake has been associated with poor dietary intake [2, 3] and weight gain [4] among young population. Fast food restaurants are becoming widespread worldwide, both in developed and even developing countries like Iran [5]. Based on reports, one-third of adolescents consume fast foods three or more times during a week [6]. Fast food contains higher levels of calorie and fat compared to the home-prepared meals [7]. Our previous studies in Isfahan, Iran, showed that the majority of students consume high servings of fast food per week [8, 9] which was related to higher dietary energy density and higher weight and waist circumference. Previous studies in Isfahan, Iran, showed that fast food consumption was associated with obesity and central adiposity [8, 9]. An important time for assessing and evaluating fast food intake and detecting the associated factors is from adolescents to younger adulthood, a high risk time for being overweight and obesity [10, 11]. Determining the factors influence on dietary intakes among adolescents, such as food preferences [4, 12], family eating patterns [13], and social norms [14, 15], could be a guide for conducting interventions aimed to adopt healthy eating behaviors. Theories and models could offer useful methods in promoting individual behavior. One of the well-known models is the theory of planned behavior (TPB) [16]. This theory could successfully predict intention and behavior in eating and has recently received great attention in determining the norms and beliefs related to fast food and snacks consumption [17–21]. TPB model has been derived from the theory of reason action (TRA) [16] and explains health behaviors that are not completely under an individual's control. According to a report [16], intention to a behavior is controlled by three concepts: attitudes (favourable or unfavourable evaluations about the behavior), subjective norms (perceived social pressure to perform behavior), and perceived behavior control (PBC) (an individual's perceptions of the ease or difficulty of performing the behavior of interest) [22, 23]. This model has been examined in several studies on eating behavior [18, 24] and has been successfully applied, in a wide range of age groups, in the field of fast food consumption [25]. One of the important age groups that are usually interested in fast food intake is adolescents [25, 26]. Attitudes, PBC, and subjective norms are major parts of TPB, and further to these main parts, TPB could include additional variables if they constitute a large proportion of variance in behavior [17]. Two additional variables could be past behavior and actual behavior control (ABC) which have been mentioned in previous studies in the field of fast food consumption [18]. TPB model could increase the possibility of predicting intention and behavior [18, 27–30]. ABC referres to those factors and resources that could affect people's ability to do a behavior [31]. Dunn et al. found that intention, ABC, and PBC explained 50% of variance in the behavior related to fast food consumption [18]. To our knowledge, no study has examined the utility of a theoretical framework of fast food consumption in an Iranian population to date. As it was mentioned previously, fast food intake is a problem in Iran [9] and we are not aware of any research based on TPB in this population for determining the beliefs and norms related to fast food intake among Iranian adolescents. Although there is a large body of the literature on TPB, according to our knowledge, it is mostly conducted in developed countries and few studies have been conducted in developing countries. In developing countries, we are faced with the burden of diseases, so we have both malnutrition and over nourishment and chronic diseases due to high calorie intake. Therefore, it is not clear that if a model is suitable in predicting some factors related to fast food intake in developed countries, it may also be successful in developing countries. So, it seems that examining the usefulness of the models in each population is necessary. The present research examines some predictors of fast food consumption using TPB model and additional variables to increase the predictability of intention and behavior among high school students in Isfahan, Iran. 

## 2. Method

### 2.1. Participants

In 2011, a sample of 521 high school students (244 boys and 277 girls) was recruited from 3 regions in Isfahan, Iran. We used a randomized stratified sampling to choose the participants of the present study. At the first stage, a systematic random-sampling procedure was used to select the schools. The schools were stratified into boys' and girls' high schools. The selection of the public high schools was proportional according to the population size. Six schools (three boys' and three girls' schools) were selected from each of the three regions in Isfahan. At the second stage, classes were selected from all grades (year of education in high school) by using a simple random-sampling design. In this way, one class was randomly selected in each of the three grades (first year, second year, and third year) in each high school. Thus, we had a total selection of at least 18 classes in each region (9 classes from the boys' schools and 9 from girls' schools). The explanations about the objectives of the present study were provided for principals of these schools. Participants completed a simple questionnaire during school hours. In total, 600 questionnaires were distributed, and 521 were returned (response rate, 86.83%). This study was approved by the Research Council and Ethics Committee of the Isfahan University of Medical Sciences.

## 3. Instrument

### 3.1. Measurement of the Constructs

First, participants completed questions related to demographic data and the TPB variables, which included attitudes, PBC, subjective norms, additional variable past behavior, and ABC. Most of the measures used within the study were developed in a previous study published by Blanchard et al. [32] based on the information obtained from initial interviews with 20 high school students.

A panel of experts, consisting of 5 experts in the fields of health behavior, education, nutrition, and a healthy eating provider with field experience in nutrition, reviewed and assessed the questions of commitment to a plan of action scale orally by evaluating the appropriateness and relevance of the items and response format. They confirmed them as being representative of the construct in order to confirm content validity of the instrument. The feedback from the panel of experts which was mostly regarding the wording and phrasing of questions was used to revise and modify the instrument.

A pilot study was conducted to examine the utility of the instruments and to identify the problems and benefits associated with the design. The questionnaire was pilot-tested with 30 students. The data were used to estimate the internal consistency of the scales, using Cronbach's coefficient alpha. The content validity of the scales was also established. 

Attitude was defined as favorable or unfavorable evaluations about the behavior. Attitude to fast food consumption was assessed as the mean of twenty questions with 5-point items including ten items for behavior beliefs and ten for outcome expectancies. Beliefs such as “*fast food tastes good*” were measured on a 5-point scale between 1 (strongly disagree) and 5 (strongly agree). Expectancies, directly related to the previous beliefs, were also measured on a 5-point scale with participants responding to a sentence like “*eating fast food frequently is good for my health*” with a score between 1 (extremely unimportant) and 5 (extremely important). Participants rated on a scale of 1–5 with a higher score indicating a more positive attitude. The reliability coefficient (alpha) for Attitude was 0.66. 

 Subjective norm was defined as perceived social pressure to perform behavior. Subjective norms were measured by four items. For example “*Most people who are important for me think that I should eat fast food regularly*” was typical of the items for this construct. Each item was presented on a 5-point scale from 1 (strongly disagree) to 5 (strongly agree), with a higher score indicating higher normative pressure. Cronbach's *α* for the overall scale was 0.71.

PBC was measured by four items. For example “*It would be impossible for me not to eat fast food regularly during the next month*” was typical of the items for this construct. Each item was presented on a 5-point scale from 1 (strongly disagree) to 5 (strongly agree), with a higher score indicating higher level of perceived control. Cronbach's *α* for the scale was 0.60. 

 ABC was assessed by two items. For example “*where I live, I have no access to fast-food outlets*.” Two items were measured on a 5-point scale between 1 (strongly disagree) and 5 (strongly agree).

 Past behavior was measured with two items, “*On average, how often do you eat fast food?*” and “*In the last week, how many times have you eaten fast food?*.” Responses were rated on a 7-point scale, ranging from 0 (never) to 7 (more than once per day) for first item and the number for fast food intake in past week for second item.

 Behavior intention was assessed using three items. For example “*given my lifestyle, it is likely that I will eat fast food regularly over the next four weeks*.” Each item was presented on a 5-point scale from 1 (strongly disagree) and 5 (strongly agree), with a higher score indicating higher intention of fast food intake. Cronbach's *α* was 0.72.

Behavior (fast food consumption) was measured with a short self-report measure of fast food consumption during one week. Eight items (sandwich, Berger-piroshky, hot dog, snack, pizza, chicken nugget, and fried chicken) were used to assess consumption at each time point. Scores from the eight items were summed to create a composite score of the fast food consumption during one week. 

## 4. Statistical Analysis

All data analyses were performed using SPSS version 14.0 with an alpha level of 0.05. To test normality of data, Kolmogorov-Smirnov test was conducted. TPB internal consistency was tested by Cronbach's alpha. Simple descriptive statistics were used to analyze the demographic data. Pearson correlation was used to examine the associations among the variables of the TPB model. We applied multiple regression analysis to predict intention and behavior with and without the additional variables. Finally, bivariate analysis and ANOVA were used to link the results of the TPB with individual characteristics of the respondents.

## 5. Results

Table 1 provides a summary of the demographic characteristics of the students in this study. There was a direct association between economic status and the amount of fast food intake in the present study (*r* = 0.235, *P* < 0.001). High levels of education of mother (*r* = 0.154, *P* < 0.001) and father (*r* = 0.138, *P* < 0.001) was positively correlated with fast food consumption. There was no significant association between parents' job and family size with fast food consumption. 

Table 2 shows means, standard deviations, range, and bivariate correlations among attitude, subjective norm, PBC, intention, behavior, past behavior, and ABC regarding fast food intake among Iranian high school adolescents. All of the TPB variables with the exception of PBC (*r* = −0.21) and actual behavior control (*r* = −0.09) were found to have significant positive correlations with intention to consume fast food (*P* < 0.01). Higher positive attitude toward fast food consumption (*r* = 0.40), and subjective norms (*r* = 0.38) were significantly associated with a stronger intention to fast food consumption (*P* < 0.001). Furthermore, those who had a higher PBC towards eating fast food had a weaker intention to fast food intake. Male students had higher perceived subjective norms than female students (*t*  (521) = −2.89, *P* < 0.05), but no gender differences were found for attitude or PBC (*P* > 0.05). 

**Figure 1:**
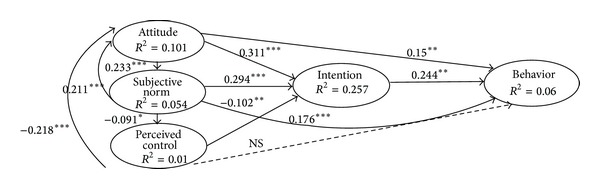
the results from the path analysis which was conducted to examine the direct and indirect effects of the TPB components on the behavior and intention.

Figure 1 shows that Attitude, subjective norm, and PBC explained 25.7% of the variance in the intention to fast food consumption. Attitude (*β* = 0.311, *P* < 0.001), subjective norm (*β* = 0.294, *P* < 0.001), and PBC (*β* = −0.102, *P* < 0.01) had the greatest influence on intention, respectively. Regarding the association of PBC with attitude and intention to fast food consumption, less PBC toward fast food consumption was associated with a stronger positive attitude and intention. Furthermore, PBC was a nonsignificant predictor of high school students' behavior (*β* = −0.01, NS). The direct and indirect effects of both attitude and subjective norms on behavior were also significant (*P* < 0.001) and subjective norms were the strongest predictive of behavior. High school students' intention was positively related to their own behavior and accounted for 6% of its variance (*P* < 0.001).

**Figure 2:**
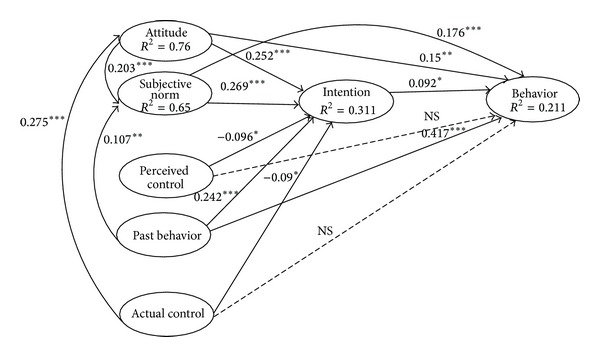
Path analysis based on the theory of planned behavior with additional variables (past behavior and ABC) to predict fast food consumption.

Figure 2 shows the TPB model with additional variables of past behavior and ABC as predictors of both intention and behavior. TPB model with additional variables explained 31.1% of the variance in the intention. Subjective norm (*β* = 0.269, *P* < 0.001), attitude (*β* = 0.252, *P* < 0.001), past behavior (*β* = 0.242, *P* < 0.001), PBC (*β* = −0.096, *P* < 0.05), and ABC (*β* = −0.090, *P* < 0.05) had the greatest influence on intention. Actual behavior control was found to be unrelated to behavior, and it was less effective than past behavior on intention (*P* < 0.05). Adding past behavior in this model significantly increased the variance in intention to 5.4% and the variance in behavior to 15.1%. Therefore, both directly and indirectly, past behavior was most predictive of behavior (*P* < 0.001). In total, 31% of the variance in intention and 21% of the variance in behavior could be predicted by the model presented in Figure 2.

## 6. Discussion 

 This study examined the applicability of TBP model and TPB model with additional variables of past behavior and ABC to determine predicting factors of fast food intake. So this study investigated the direct and indirect effects of TPB constructs on intention and behavior. The major finding was that TPB with additional past behavior and ABC had higher power compared to the classic TPB framework. In TPB model, the theory explained 25.7% of the intention. This was lower than the reported value from other study which showed that attitude, subjective norms, and PBC explained 50% of the variance in intention to fast food consumption in an Australian sample [18]. We found that intention to consume fast food was predicted by attitude (direct effect), subjective norm, and PBC (negative relationship), either directly or indirectly. Interestingly, while some studies suggest that subjective norms [17, 18] are better predictors of intention to consume fast food than attitude, in the present study, attitude was a stronger predictor of intention than subjective norms and PBC in the TPB model, but in the TPB with additional variable model subjective norms were the strongest predictor. Similar findings reported that attitude toward snacking was the strongest predictor of intentions, followed by subjective norm and PBC [27]. In this study, a strong subjective norm was related to decreased perception of controllability to consume fast food, thus, indicating the importance of social influence in fast food consumption. Our findings also indicated that intention was a proximal predictor of behavior. Consistent with the results of Collins and Mullan's study [27] intention was found to be a significant predictor of behavior, accounting for 28.8% of variance in snacking among undergraduate psychology students from an Australian University. This finding showed that the interventions to increase students' motivation for reducing the frequency of fast food consumption may need to focus on creating more positive attitudes regarding healthy eating, social norms, and increasing PBC to limit fast food consumption. Consequently, greater understanding of these determinants is likely to help in the development and implementation of future intervention for students, a group that consumes fast food more frequently than other age groups [19, 33, 34] emphasizing supporting peers to engage in healthy eating behaviors. 

The TPB model with additional behavior in the present study showed that the model was more capable of explaining variation in behavior. Another noteworthy finding in the path analysis was the direct and indirect effect of past behavior. Past behavior had stronger direct and indirect effect on fast food consumption and significantly increased the amount of explained variance in both intention and behavior in our study. In the previous studies [27], past behavior improved the prediction of intention and behavior and it accounted for 13.6% of variance in intention and 21.5% of variance in snacking. The path model in the present study showed direct effect of ABC and indirect effect of behavior on students' intention to consume fast food. High school students in Isfahan were more likely to report positive attitude toward fast food intake and more likely to consume fast food with their friends and parents. Healthy eating for Iranian students should also aim at changing habitual behaviors. The results indicated that the older 15 to 18 year adolescents who reported the intention to consume fast food had a less PBC and actual control. Conversely, Collins and Mullan reported positive correlations between PBC and snaking intention [27]. All variables had a direct effect on behavior except for PBC and ABC that exert indirect effects on behavior in the present study. Although previous studies in Iran emphasized on the role of dietary intake on the risk of chronic diseases [35] as well as nutrients deficiency [36], we are not aware of studies mostly focus on the field of specific determinants such as norms and beliefs in Iran. 

 Seo and Lee examined fast food consumption status among middle school students and explored factors influencing fast food consumption using TPB. TPB effectively explained fast food consumption behaviors with relatively high R2 around 0.6. It has been suggested that higher intention, perceived behavior control, attitude, and subjective norm led to higher rates of fast food consumption behaviors among middle school students in Seoul [19]. The present study, however, focused on adolescents in Iran. There might be some differences between the results in the researches in Middle Eastern countries and other countries in other parts of the world. Thus, the discrepancy between our findings and other studies findings [17–19] may be due to differences in the age, cultural context, or health motivations. In this study, both models performed better in explaining the intentions than fast food consumption. It is suggested that future studies focus on TPB model with additional variables to reduce fast food consumption among children and adolescents in Iran. 

## 7. Limitations

The strengths and limitations of this study should be noted. Strengths of the current study were a novel investigation regarding the predictors of fast food intake of high school students in Isfahan, Iran that identified possible avenues for interventions. Beaulieu and Godin emphasized on the importance of using TPB to develop nutrition-based interventions for encouraging high school students to stay in school for lunch instead of eating in fast food restaurants [37]. One limitation of the current study relates to the potentially biased sample of high school students aged 15–18 years. We had access to just this age group among adolescents and according to our hypothesis that that fast food consumption is a problem among adolescents, we just focused on adolescents in this study. However, researches have shown that this age group had higher intake of fast foods [38]. So, we should keep in our mind that the results of the present study could be specified for adolescents. Future research in Iran should involve another age group and obtain more detailed dietary information of various groups to better understand the relationship between beliefs and fast food consumption. In addition, self-report data and the cross-sectional design were used for this study. This study is limited due to its cross-sectional nature. The present study basically confirmed the applicability of the TPB to adolescent students in Isfahan, Iran, and the effect of past behavior and ABC on their intention to and behavior of fast food intake. Thus, further studies could examine the effectiveness of various strategies in altering the psychological determinant of fast food intake among Iranian students. 

## 8. Conclusion

Findings indicate that TPB is useful for predicting factors directly and indirectly related to fast food consumption among high school students in Isfahan by comparing two models of TPB. The study was the first to use the TPB to predict fast food consumption in an adolescent population in a developing country, Iran. Subjective norms in TPB model and past behavior in TPB model with additional variables (past behavior and actual behavior control) were the most powerful predictors of fast food consumption. This study suggests that a higher social pressure from peers or family and higher past behavior had the strong influence of fast food consumption. This difference between studies in other countries may be attributed to sociocultural differences and differing behavioral patterns in adolescents towards fast food consumption. It is concluded that the TPB may be used in developing countries, like Iran, as a framework for planning intervention programs in order to predict and improve the healthy eating behavior of students. Among Iranian adolescents, eating fast food may be part of their lifestyles; thus, our results suggest that intervention programmers should focus on the beliefs, social norms, and past behavior based on PBT model to help adolescents in deciding how often to eat fast food, controlling their energy intakes and improving their overall diet quality.

## Figures and Tables

**Table 1:** Demographic characteristics of the students.

Total students	521
Mean age ± SD	16.28 ± 0.8
Age (years)	
15	115 (22.1)
16	184 (35.3)
17	180 (34.5)
18	42 (8.1)
Gender	
Male	277 (53.2)
Female	244 (46.8)
Year of education in high school	
First year	202 (38.8)
Second year	173 (33.2)
Third year	146 (28)
Father's education (%)	
Illiterate	16 (3.1)
Primary (1–9 years)	73 (14)
High school (10-11 years)	102 (19.6)
Diploma (12 years)	215 (41.3)
College/university education	106 (20.4)
Mother's education (%)	
Illiterate	13 (2.5)
Primary (1–9 years)	94 (18)
High school (10-11 years)	112 (21.5)
Diploma (12 years)	217 (41.7)
College/university education	81 (15.5)
Father's job (%)	
Worker	74 (14.2)
Employee	135 (25.9)
Teacher	13 (2.5)
Out of job	243 (46.6)
Retired	27 (5.2)
Others	18 (3.5)
Mother's job (%)	
Housewives	440 (84.5)
Employee	23 (4.4)
Teacher	26 (5)
Out of job	13 (2.5)
Others	9 (1.7)
Family size	
3 persons	46 (8.8)
4 persons	236 (45.3)
5 persons	144 (27.6)
6 persons	70 (13.4)
Household income	
<300000 Rial	105 (20.2)
300000–600000 Rial	183 (35.1)
600000–1000000 Rial	104 (20)
>1000000 Rial	52 (10)
Socioeconomic status	
Low	11 (2.1)
Low-middle	49 (9.4)
Middle	267 (50.7)
Upper-middle	167 (32.1)
High	26 (5)

**Table 2:** Means, standard deviations, range, bivariate correlations among attitude, subjective norm, PBC, intention, behavior, past behavior, and ABC regarding fast food intake among Iranian high school adolescents.

Variable	Mean	SD	Range	2	3	4	5	6	7
(1) Attitude	26.35	12.18	(10–50)	0.23**	−0.24**	0.40**	0.19**	−0.06**	0.27**
(2) Subjective norm	9.86	3.21		(4–20)	−0.10*	0.38**	0.21**	−0.08	0.16**
(3) PBC	14.67	2.93			(4–20)	−0.21**	−0.05	0.11*	0.09*
(4) Intention	7.51	2.24				(3–15)	0.24**	−0.09*	0.36**
(5) Behavior	3.37	4.58					(0–31.1)	−0.05	0.45**
(6) ABC	6.47	1.53						(2–10)	−0.02
(7) Past behavior									(0–11)

*P < 0.05, **P < 0.01.

## References

[1] Hastert TA, Babey SH, Diamant AL, Brown ER (2005). More California teens consume soda and fast food each day than five servings of fruits and vegetables. *Policy Brief (UCLA Center for Health Policy Research)*.

[2] Bowman SA, Gortmaker SL, Ebbeling CB, Pereira MA, Ludwig DS (2004). Effects of fast-food consumption on energy intake and diet quality among children in a national household survey. *Pediatrics*.

[3] French SA, Story M, Neumark-Sztainer D, Fulkerson JA, Hannan P (2001). Fast food restaurant use among adolescents: associations with nutrient intake, food choices and behavioral and psychosocial variables. *International Journal of Obesity*.

[4] Niemeier HM, Raynor HA, Lloyd-Richardson EE, Rogers ML, Wing RR (2006). Fast food consumption and breakfast skipping: predictors of weight gain from adolescence to adulthood in a nationally representative sample. *Journal of Adolescent Health*.

[5] Sadrzadeh Yeganeh H, Alavi AM, Dorostymotlagh AR, Mahmoodi M, Jarollahi N, Chamari M (2007). Relationship between obesity and nutritional behavior among high-school girls in Kerman. *Payesh*.

[6] Bauer KW, Larson NI, Nelson MC, Story M, Neumark-Sztainer D (2009). Socio-environmental, personal and behavioural predictors of fast-food intake among adolescents. *Public Health Nutrition*.

[7] Guthrie JF, Lin B-H, Frazao E (2002). Role of food prepared away from home in the American diet, 1977-78 versus 1994-96: changes and consequences. *Journal of Nutrition Education and Behavior*.

[8] Kelishadi R, Sadry GH, Hashemi Pour M (2003). Lipid profile and fat intake of adolescents: Isfahan healthy heart program—heart health promotion from children. *Koomesh Journal of Semnan University of Medical Sciences*.

[9] Rouhani MH, Mirseifinezhad M, Omrani N, Esmaillzadeh A, Azadbakht L (2012). Fast food consumption, quality of diet, and obesity among Isfahanian adolescent girls. *Journal of Obesity*.

[10] Gordon-Larsen P, Adair LS, Nelson MC, Popkin BM (2004). Five-year obesity incidence in the transition period between adolescence and adulthood: The National Longitudinal Study of Adolescent Health. *American Journal of Clinical Nutrition*.

[11] Harris KM, Gordon-Larsen P, Chantala K, Udry JR (2006). Longitudinal trends in race/ethnic disparities in leading health indicators from adolescence to young adulthood. *Archives of Pediatrics & Adolescent Medicine*.

[12] Woodward DR, Boon JA, Cumming FJ, Ball PJ, Williams HM, Hornsby H (1996). Adolescents’ reported usage of selected foods in relation to their perceptions and social norms for those foods. *Appetite*.

[13] Neumark-Sztainer D, Hannan PJ, Story M, Croll J, Perry C (2003). Family meal patterns: associations with sociodemographic characteristics and improved dietary intake among adolescents. *Journal of the American Dietetic Association*.

[14] Story M, Neumark-Sztainer D, French S (2002). Individual and environmental influences on adolescent eating behaviors. *Journal of the American Dietetic Association*.

[15] French SA, Story M, Jeffery RW (2001). Environmental influences on eating and physical activity. *Annual Review of Public Health*.

[16] Ajzen I (1991). The theory of planned behavior. *Organizational Behavior and Human Decision Processes*.

[17] Dunn KI, Mohr PB, Wilson CJ, Wittert GA (2008). Beliefs about fast food in Australia: a qualitative analysis. *Appetite*.

[18] Dunn KI, Mohr P, Wilson CJ, Wittert GA (2011). Determinants of fast-food consumption. An application of the theory of planned behaviour. *Appetite*.

[19] Seo H-S, Lee S-K, Nam S (2011). Factors influencing fast food consumption behaviors of middle-school students in Seoul: an application of theory of planned behaviors. *Nutrition Research and Practice*.

[20] Branscum P, Sharma M (2013). Comparing the utility of the theory of planned behavior between boys and girls for predicting snack food consumption: implications for practice. *Health Promotion Practice*.

[21] Branscum P, Sharma M (2011). Using the theory of planned behavior to predict two types of snack food consumption among midwestern upper elementary children: implications for practice. *International Quarterly of Community Health Education*.

[22] Ajzen I, Fishbein M, Albarracín D, Johnson BT, Zanna MP (2005). The influence of attitudes on behaviour. *Handbook of Attitudes and Attitude Change: Basic Principles*.

[23] Ajzen I, Fishbein M (1980). *Understanding Attitudes and Predicting Social Behavior*.

[24] Ajzen I, Madden TJ (1986). Prediction of goal-directed behavior: attitudes, intentions, and perceived behavioral control. *Journal of Experimental Social Psychology*.

[25] de Bruijn GJ, Kroeze W, Oenema A, Brug J (2008). Saturated fat consumption and the theory of planned behaviour: exploring additive and interactive effects of habit strength. *Appetite*.

[26] Wong CL, Mullan BA (2009). Predicting breakfast consumption: an application of the theory of planned behaviour and the investigation of past behaviour and executive function. *British Journal of Health Psychology*.

[27] Collins A, Mullan B (2011). An extension of the theory of planned behavior to predict immediate hedonic behaviors and distal benefit behaviors. *Food Quality and Preference*.

[28] Sheeran P (2002). Intention-behavior relations: a conceptual and empirical review. *European Review of Social Psychology*.

[29] Norman P, Armitage CJ, Quigley C (2007). The theory of planned behavior and binge drinking: assessing the impact of binge drinker prototypes. *Addictive Behaviors*.

[30] Smith JR, Terry DJ, Manstead ASR, Louis WR, Kotterman D, Wolfs J (2007). Interaction effects in the theory of planned behavior: the interplay of self-identity and past behavior. *Journal of Applied Social Psychology*.

[31] Ajzen I Theory of planned behavior. http://people.umass.edu/aizen/tpb.html.

[32] Blanchard CM, Kupperman J, Sparling PB (2009). Do ethnicity and gender matter when using the theory of planned behavior to understand fruit and vegetable consumption?. *Appetite*.

[33] Satia JA, Galanko JA, Siega-Riz AM (2004). Eating at fast-food restaurants is associated with dietary intake, demographic, psychosocial and behavioural factors among African Americans in North Carolina. *Public Health Nutrition*.

[34] Bowman SA, Vinyard BT (2004). Fast food consumption of US adults: impact on energy and nutrient intakes and overweight status. *Journal of the American College of Nutrition*.

[35] Azadbakht L, Esmaillzadeh A (2008). Dietary and non-dietary determinants of central adiposity among Tehrani women. *Public Health Nutrition*.

[36] Mirmiran P, Azadbakht L, Azizi F (2006). Dietary diversity within food groups: an indicator of specific nutrient adequacy in Tehranian women. *Journal of the American College of Nutrition*.

[37] Beaulieu D, Godin G (2012). Development of an intervention programme to encourage high school students to stay in school for lunch instead of eating at nearby fast-food restaurants. *Evaluation and Program Planning*.

[38] Briefel RR, Johnson CL (2004). Secular trends in dietary intake in the United States. *Annual Review of Nutrition*.

